# Arabidopsis C-Terminal Domain Phosphatase-Like 1 Functions in miRNA Accumulation and DNA Methylation

**DOI:** 10.1371/journal.pone.0074739

**Published:** 2013-09-18

**Authors:** In Sil Jeong, Emre Aksoy, Akihito Fukudome, Salina Akhter, Akihiro Hiraguri, Toshiyuki Fukuhara, Jeong Dong Bahk, Hisashi Koiwa

**Affiliations:** 1 Division of Applied Life Science (BK21 Program), Graduate School of Gyeongsang National University, Jinju, Gyeongsangnam-do, Korea; 2 Department of Horticultural Sciences, Texas A&M University, College Station, Texas, United States of America; 3 Department of Applied Biological Sciences, Tokyo University of Agriculture and Technology, Fuchu, Tokyo, Japan; Kansas State University, United States of America

## Abstract

Arabidopsis CTD-PHOSPHATASE-LIKE 1 (CPL1) is a protein phosphatase that can dephosphorylate RNA polymerase II C-terminal domain (CTD). Unlike typical CTD-phosphatases, CPL1 contains a double-stranded (ds) RNA-binding motif (dsRBM) and has been implicated for gene regulation mediated by dsRNA-dependent pathways. We investigated the role of CPL1 and its dsRBMs in various gene silencing pathways. Genetic interaction analyses revealed that *cpl1* was able to partially suppress transcriptional gene silencing and DNA hypermethylation phenotype of *ros1* suggesting CPL1 is involved in the RNA-directed DNA methylation pathway without reducing siRNA production. By contrast, *cpl1* reduced some miRNA levels at the level of processing. Indeed, CPL1 protein interacted with proteins important for miRNA biogenesis, suggesting that CPL1 regulates miRNA processing. These results suggest that CPL1 regulates DNA methylation via a miRNA-dependent pathway.

## Introduction

Plant gene expression is transcriptionally and post-transcriptionally regulated by a population of small RNAs. The small RNA biogenesis involves diverse factors that determine levels of specific type of small RNA [1]–[4]. Micro RNAs (miRNA) are 21-base long and their biogenesis starts from the transcription of *MIR* genes by RNA polymerase II (Pol II). The resulting pri-miRNAs are processed by a complex containing Dicer-like 1 (DCL1) [5], HYPONASTIC LEAVES1 (HYL1) [6], [7] and SERRATE (SE) [8] leading to the production of a miRNA-miRNA* duplex [9], [10]. siRNAs are generated from long dsRNAs, which are derived from transcription of inverted repeats, transposable elements, and conversion of single stranded RNAs by RNA-dependent RNA polymerases (RDRs) [11], and subsequent processing by various DCL proteins [12].

Among various roles of small RNAs, siRNA can promote DNA methylation via the canonical RNA-mediated DNA methylation (RdDM) pathway [13]. siRNAs bind to ARGONAUTE4, which in turn forms a complex with various factors, such as RNA polymerase V and KTF [14]–[17], required for defining RdDM targets loci, where DRM2 is recruited to catalyze DNA methylation [18], [19]. Other factors such as RNA polymerase II (pol II) [20], mediator [21], splicing machineries [22], and DCL1 [23], [24] also contribute to the establishment of the DNA methylation. Steady-state level of DNA methylation is determined by antagonizing activities of methylation and demethylation. In plants, some DNA demethylations are mediated by ROS1, which cleaves the DNA backbone to remove methyl-cytosine from the DNA double strand [25]. The *ros1* plants exhibit DNA hypermethylation and enhanced transcriptional gene silencing (TGS) in various loci including promoter region of a transgene *RD29A-LUC* and its endogenous counterpart [25].

We have previously identified an Arabidopsis C-terminal domain (CTD)-phosphatase-like 1 (CPL1) by forward genetic screening using the *RD29A-LUC* reporter gene [26], [27]. *cpl1* causes hyperactivation of *RD29A-LUC,* opposite to *ros1* in the same background [25]. CPL1 and its paralog CPL2 can dephosphorylate CTD of the pol II largest subunit specifically at the Ser5 of heptad repeat sequence (Y^1^S^2^P^3^T^4^S^5^P^6^S^7^) suggesting their role in transcription elongation and mRNA maturation [28], [29]. CPL1 regulates gene expression in various biological processes, including osmotic stress and iron deficiency [26], [27], [30]. However, little is known about how CPL1 regulates gene expression. Here we report roles of CPL1 in small RNA-mediated gene expression.

## Materials and Methods

Primer sequences were listed in Table S1.

### Plant Materials, Growth Condition, and Stress Treatments

*Arabidopsis thaliana cpl1-2* (formerly *fry2-1*), and *ros1–1* mutants [25]–[27] in ecotype C24 carrying an RD29A*-LUC* reporter gene [31], and L1 line carrying post-transcriptionally silenced *35S-GUS* reporter (ecotype Columbia) [32] were used in this study. *cpl1-2* transformed with *gCPL1-FLAG* transgene will be described elsewhere. Plants were grown on agar plates containing 1/2×Murashige and Skoog salts and 1% sucrose. Cold and ABA treatments were applied to 2-week-old plants as described [26]. Arabidopsis cell culture was induced and maintained as described [33]. Heat treatment was applied as described [34].

### Reporter Gene Assays

In vivo luciferase activity was documented as described [26]. Cold stress (0°C, 48 hr) treated plants were sprayed with luciferin solution (0.01% TritonX-100, 1 mM Luciferin) and kept for 5 min in the dark. Image acquisition and processing were performed with Electron Multiplying Charge-Coupled Device camera (Cascade II, Photometrics) and the WinView software (Roper Scientific). β-glucuronidase assay was performed as described [35]. Plants were transferred into staining solution that contains 0.5 mM K_3_Fe(CN)_6_, 0.5 mM K_4_Fe(CN)_6_, 0.3% v/v Triton X-100 and 2 mM X-gluc (Sigma-Aldrich), vacuum infiltrated for 5 min, and incubated at 37°C overnight. After staining, tissues were washed with 70% ethanol.

### RNA Analysis

The total RNA and small RNA were extracted from 10-day-old seedlings using E.Z.N.A.® PF Micro RNA Kit and E.Z.N.A. miRNA Isolation Kit (Omega Biotek), respectively. For RT-qPCR analysis, small RNA samples were converted to cDNA using NCode™ miRNA First-Strand cDNA Synthesis Kit (Life Technologies) and were subjected to qPCR analysis. qPCR were performed and analyzed as described [30] using the primers listed in Table S1.

Small RNA Northern blotting analysis was performed as described [1]. The small RNA samples (80 µg) were resolved on 17% polyacrylamide gel containing 7 M Urea in 1× TBE buffer and were transferred onto nylon filters. The filters were hybridized by ^32^P-labeled small RNA probes at 42°C in PerfectHyb™ Plus buffer (SIGMA). The filters were then washed three times with 2× SSC, 0.1% SDS at 42°C for 20 min and exposed to X-ray film. The sequence of oligonucleotide probes used for small RNA Northern blotting analysis are listed in Table S1.

### DNA Methylation Analysis

DNA methylation analysis EpiTect® Bisulfite Kit (QIAGEN) was performed as described [25]. Endogenous and transgene RD29A promoter fragments were amplified by PCR using the bisulfite-treated genomic DNA samples (2 µg) as template. The PCR products of two amplified promoters were cloned into pGEM-T easy vector (Promega) and 15 individual clones were sequenced for each sample. The primers used for bisulfite sequencing are listed in Table S1. PCR-based DNA methylation assays were performed as described [1], [36]. Genomic DNA (500 ng) was digested with the methylation-sensitive restriction enzyme *Hae* III overnight at 37°C or the methylated DNA-digesting enzyme *McrBC* for 1 hr at 37°C. The digested DNA was used to amplify the RdDM targets, including *AtSN1*, *AtGP1*, and *AtMU1*. The undigested genomic DNA was simultaneously amplified as control. PCR conditions were 3 min at 94°C, followed by 30 cycles of 94°C for 30 s, 53°C for 30 s, and 72°C for 40 s, and a final extension step at 72°C for 10 min. Three independent experiments were performed for *AtMU1* and two independent experiments were performed for *AtSN1* and *AtGP1*. PCR-based DNA methylation assays was performed using specific primers listed in Table S1.

### Co-immunoprecipitation Assay

*Arabidopsis* calli expressing *gCPL1-FLAG* were homogenized in an extraction buffer containing 100 mM Tris–HCl pH 7.5, 2 mM EDTA, 25% glycerol, 2 mM DTT, 1 mM PMSF, 100 µg/l DNase, 50 µg/ml RNase, and 1× complete protease inhibitor cocktail. Protein extracts were centrifuged twice at 16,000 rpm at 4°C for 15 min, and protein concentration in the supernatant was determined by Bradford reagent assay. The cleared protein extract (110 µg protein) was incubated with 5 µl anti-FLAG antibody (or anti-HYL1) for 5 min and, the immunocomplex was precipitated after incubation with 7 µl of protein A agarose resin overnight at 4°C with gently shaking and collected by centrifugation. After washing the immunoprecipitated proteins by TTBS were subjected to SDS-PAGE and specific proteins were detected by Western blotting using specific antibodies (anti-HYL1 and anti-FLAG).

### Bimolecular Fluorescence Complementation (BiFC) Assay

cDNA fragments encoding a full-length CPL1 and a full-length HYL1 were cloned into BiFC vectors [37] to produce pCPL1-nYFP and pHYL1-cYFP. The transformation DNA mixtures contained the indicated combinations of 5 µg of each DNA preparation. Polyethylene glycol-mediated transformation of *Arabidopsis* protoplasts were performed as described [28].

### Luciferase Complementation Imaging (LuCI) Assay

LuCI was performed as described [38]. *CPL1*, *CPL1^1–714^*, *CPL1^699–967^*, *HYL1^1–223^* and *SE* fragments were cloned in pDONRzeo (Life Technologies) by Gateway BP reaction and then transferred into pDEST-NLUC^GW^ or pDEST-CLUC^GW^ [38] by Gateway LR reaction (Life Technologies). Resulting NLUC/CLUC constructs and a 35S-P19 construct (provided by Dr. Baulcomb) were introduced into *Agrobacterium tumefaciens* GV3101 cells [39].

To test interactions, GV3101 cells carrying the various NLUC/CLUC constructs were prepared as follows. Cells grown on solid LB medium supplemented with 50 µg/ml kanamycin were inoculated in 10 ml of liquid LB kanamycin medium. After 20 h incubation, cells were harvested by centrifugation at 4000 rpm for 10 min and re-suspended in fresh activation medium containing 10 mM MES/KOH (pH 5.6), 10 mM MgCl_2_ and 150 µM acetosyringone. Cell suspensions were mixed to achieve a final OD_600_ of 0.4 for NLUC/CLUC constructs and 0.15 for the P19 helper strain, respectively. The 100 µl of NLUC, CLUC and P19 cell suspension mixtures were infiltrated into leaves of 4- to 7-week-old *Nicotiana benthamiana* plants. Luminescence images were taken 3d after infiltration. Leaves were infiltrated with luciferin solution (10 mM MES/KOH, pH 5.6, 10 mM MgCl_2_ and 100 µM luciferin) and images were acquired using an EMCCD camera and processed by WinView software.

### Yeast two-hybrid Assays

For the yeast two-hybrid analysis, *CPL1*, *HYL1* and *SE* fragments were amplified by PCR and cloned into pDONRzeo by the Gateway BP reaction. Gateway compatible two-hybrid vectors, pBUTE^GW^ and pGAD^GW^, were prepared by inserting Gateway cassette A (Life Technologies) into the SmaI site of pBute [40] or pGAD.c1 [41], and were used to clone *CPL1, HYL1* and *SE* fragments by Gateway LR reactions. Lithium acetate-mediated transformation of yeast strain PJ69-4A was performed as described [42]. After transformation, yeast were plated on synthetic dropout media (SD) composed of nitrogen base, 2% glucose and a dropout supplement without uracil and leucine (-UL) and incubated at 28°C for 48 hr. 2×10^5^ cells of colonies growing on SD/−UL and their diluted cells (2×10^4^ cells) were transferred onto SD composed of nitrogen base, 2% glucose, a dropout supplement without uracil, leucine, histidine and adenine (-ULHA) and incubated at 28°C for 48 h.

## Results and Discussion

### The *cpl1* Suppresses Transcriptional Gene Silencing by *ros1*

**Figure 1:**
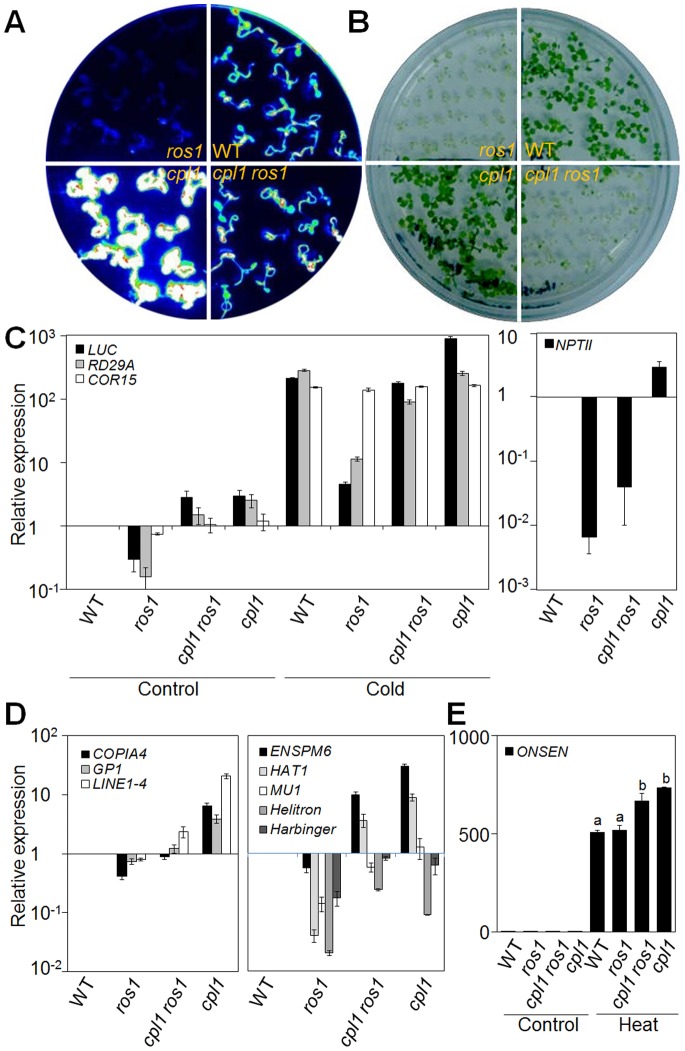
*cpl1* partially suppresses *ros1* gene silencing phenotype. *RD29A-LUC* luminescence image after cold treatment (0°C, 48 hr, A) and photograph showing kanamycin resistance (50 µg/ml, B) of WT, *ros1*, *cpl1 ros1,* and *cpl1*. (C) RT-qPCR analysis of the transcript levels of *RD29A-LUC*, endogenous *RD29A* and *COR15A* (left) and *NPT II* (right). (D) RT-qPCR analysis of the transcript levels of retrotransposons (left) and DNA transposons (right). Bars indicate standard errors of the mean (SEM) from three biological replicates. (E) RT-qPCR analysis of the transcript levels of *ONSEN*. Bars indicate standard errors of the mean (SEM) of two biological replicates. Different letters show significant differences (*p*<0.05, one-way ANOVA followed by Tukey’s HSD post hoc test).

Overexpression of the cold-stress-inducible RD29A*-LUC* reporter is a hallmark phenotype of *cpl1* mutants. The expression level of RD29A*-LUC* is controlled by many factors including transcriptional gene silencing via RdDM. Since CPL1 has dsRBMs at its C-terminus, we tested if CPL1 is involved in dsRNA mediated gene regulations, i.e., transcriptional and post-transcriptional gene silencing (TGS and PTGS). The *cpl1-2* line (hereafter referred as *cpl1*) was crossed with RD29A*-LUC ros1–1* plants (hereafter referred as *ros1*) and with 35S-GUS L1 plants, representative systems to test TGS and PTGS in plants, respectively. As shown in Figures 1A to C, the expression levels of RD29A*-LUC* and *35S-NPTII* were substantially decreased in *ros1* plants, resulting in lack of cold-induced luminescence and kanamycin sensitivity. Interestingly, in *cpl1 ros1* double mutants, expression of RD29A*-LUC* but not *35S-NPTII* and kanamycin resistance phenotype was partially restored. These results indicate that *cpl1* suppresses gene silencing caused by *ros1*, but in a target specific manner.

To test the specificity of antagonistic interaction between *ros1* and *cpl1,* expression levels of various transposable elements that are targets of gene silencing in *ros1* were analyzed. Consistent with the previous report [43], *ros1* downregulates expression of both transposons (Figure 1D, right) and retrotransposons (Figure 1D, left) tested. Individual transposable elements produced a unique expression profile in the different genetic backgrounds tested. In general, retrotransposons were down and up-regulated in *ros1* and *cpl1*, respectively, and show intermediate levels in *cpl1 ros1*. This is indicative of an antagonistic effect of CPL1 and ROS1. Interestingly, expression of *ONSEN*, a heat inducible *copia-*like retrotransposon [34], was enhanced in *cpl1* but was not affected in *ros1* (Figure 1E). By contrast, both increases and decreases in *cpl1* were observed for transcripts encoded by DNA transposons (Figure 1D, right). *cpl1 ros1* showed an expression profile similar to that of *cpl1,* indicating likely epistasis of *cpl1* over *ros1*. These results indicate that the effect of *cpl1* is unique to individual genes, and *RD29A-LUC* transgene and retrotransposons are similarly regulated by CPL1.

The role of CPL1 in PTGS was evaluated using the L1 line, which carries a *35S-GUS* transgene that undergoes PTGS [32], [44]. Since GUS activity staining of L1 wild type and L1 *cpl1* did not differ (Figure S1), we concluded that CPL1 is not necessary for PTGS of L1 transgene.

### DNA Methylation Level at RdDM Target Loci is Reduced in *cpl1*

**Figure 2:**
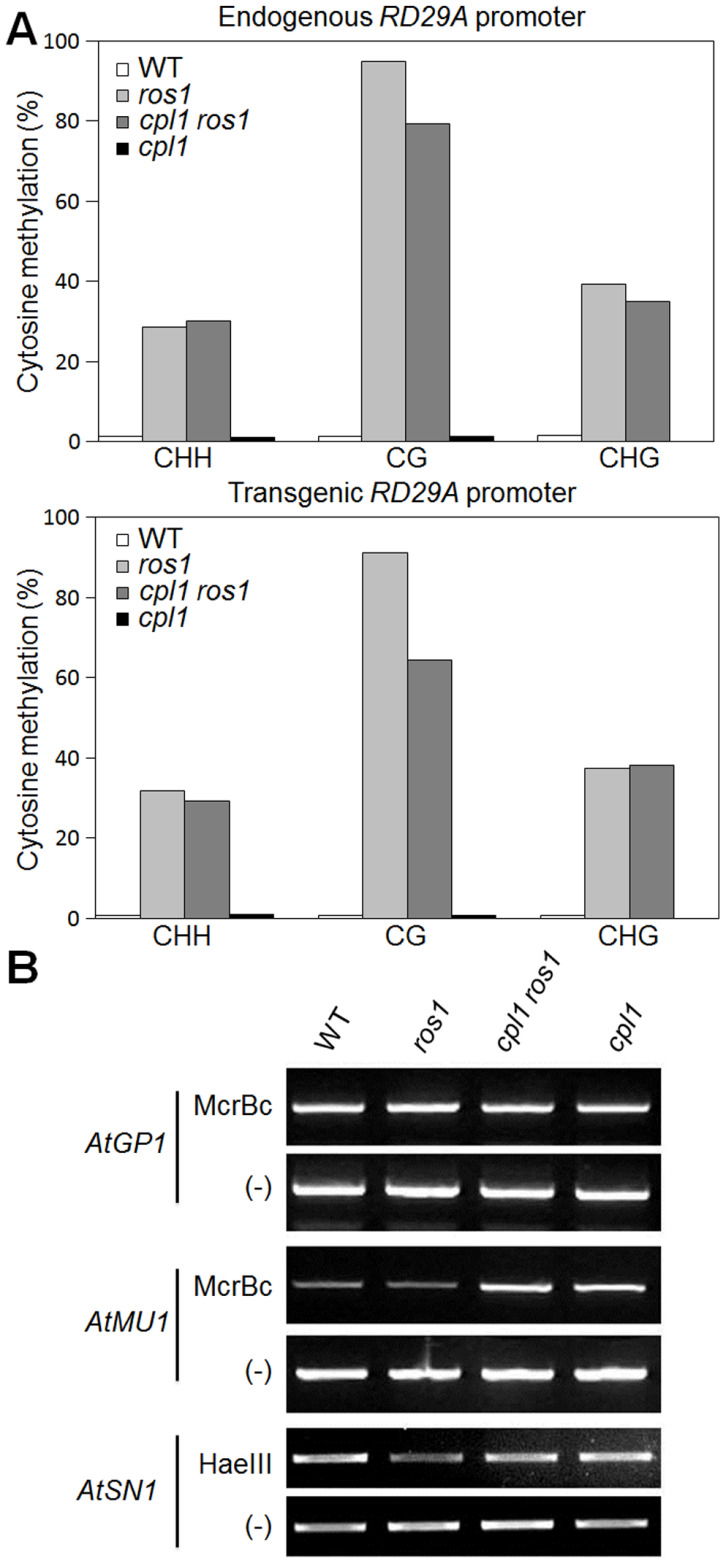
The *cpl1* mutation influences DNA methylation. (A) Bisulfite sequencing results of endogenous *RD29A* and *RD29A-LUC* transgene promoters. The ratio of cytosine methylation in percentage was determined at CG, CHG, and CHH sites on endogenous (left) and transgenic (right) *RD29A* promoters. H represents A, T, or C. (B) PCR-based cytosine methylation assay on RdDM target loci using methylation-sensitive enzymes. The amplifications using undigested DNA templates (-) were used as controls.

In *ros1, RD29A-LUC* is silenced by DNA methylation. To test if *cpl1 ros1* restores RD29A*-LUC* expression by decreasing DNA methylation levels, we performed bisulfide sequencing analysis of the endogenous and the transgene *RD29A* promoters. As shown in Figure 2A, extensive cytosine methylation of both *RD29A* promoters in all sequence contexts (CG, CHG, and CHH; H represents A, T, or C) were observed in *ros1.* In *cpl1 ros1*, the CG methylation level was reduced 25% in transgene *RD29A* promoter and 16% in endogenous *RD29A* promoter. The partial reversion of DNA methylation is consistent with the partial release of gene silencing at these loci.

DNA methylation of *RD29A* promoter occurs via RdDM. We therefore tested DNA methylation levels of other RdDM targets, i.e., *AtMU1, AtGP1* and *AtSN1* using PCR-based assays (Figure 2B). As reported previously, a DNA transposon *AtMU1* is heavily methylated in wild type and *ros1.* However, consistent with the higher expression levels, *cpl1* and *cpl1 ros1* mutants showed decrease in *AtMU1* methylation level. By contrast, no alteration was detected in methylation levels of *AtGP1 and AtSN1* in *cpl1.* Together, these results indicate that *cpl1* mutation affect DNA methylation of some but not all RdDM targets.

### *cpl1* Affects Small RNA Levels

**Figure 3:**
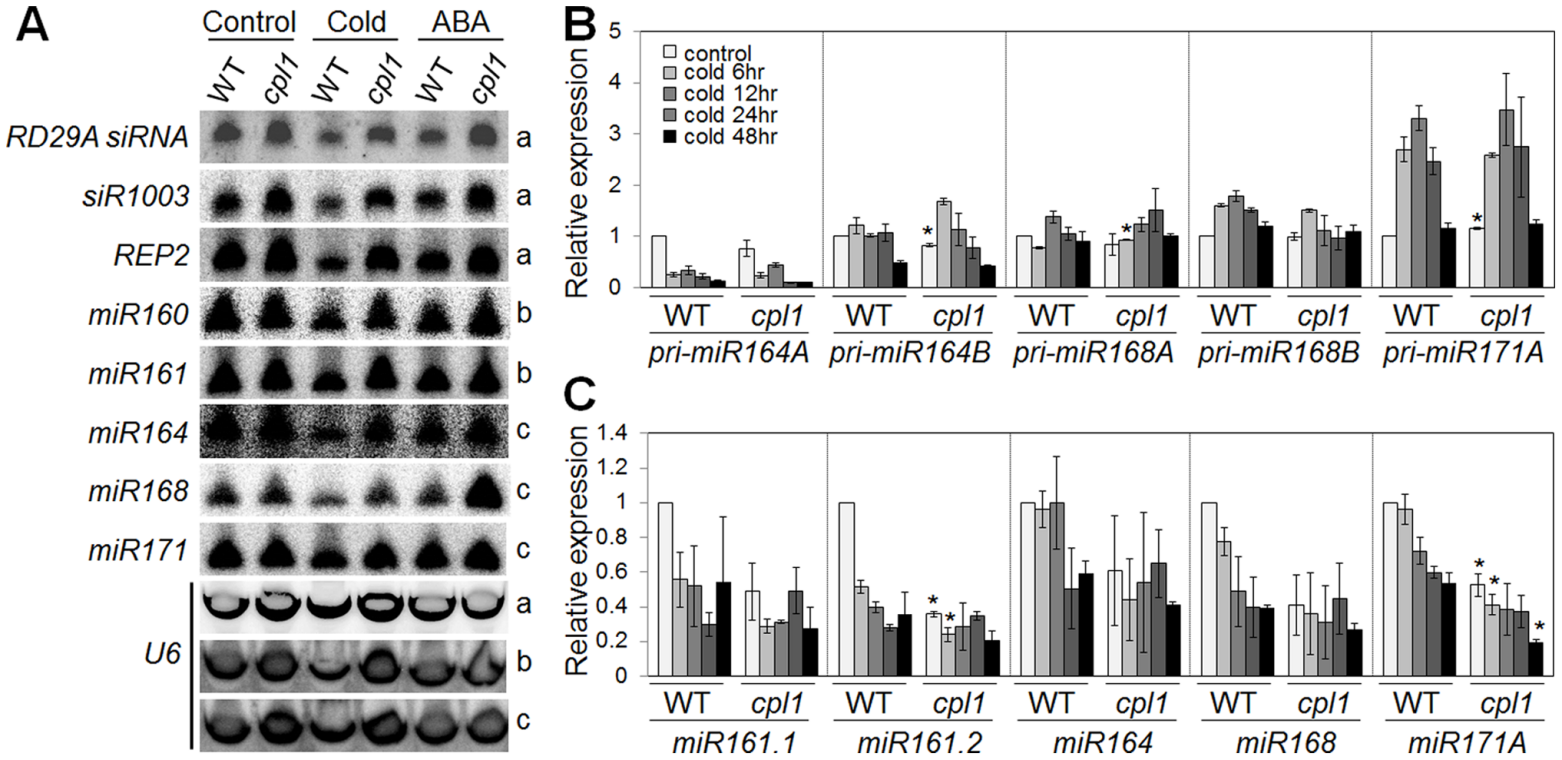
Differential accumulation of small RNAs in *cpl1* under cold stress. (A) Northern blotting analyses of small RNAs in WT and *cpl1* under stress conditions. Two-week-old plants were treated with cold (0°C) for 48 h or with 100 µM ABA for 3 h. *U6* was used as loading controls. The different letters on the right side indicate independently prepared membrane blots. (B and C) Time course analyses of cold response of select pri-miRNA (B) and mature miRNA (C) levels by RT-qPCR. Bars indicate standard errors of the mean (SEM) of two biological replicates. *p<0.05, Student’s *t*-test between mean values of *cpl1* and Col-0 for the same conditions.

Since silencing of RD29A/*RD29A-LUC* in *ros1* is dependent on production of RD29A promoter siRNA, we tested if *cpl1* decreases RD29A promoter siRNA level during cold stress and ABA treatment (hormonal inducer of RD29A) by Northern blotting (Figure 3A). Unexpectedly, the siRNA level was slightly decreased in cold-treated wild type but not in *cpl1.* Similar decreases were observed only in wild type for several additional small RNAs, such as siRNA1003 and REP2, and miRNA160, 161, 164, 168, and 171 (Figure 3A) but not other small RNAs tested (miR157, 159, 167, 173, 390, and TAS1, 2, and 3, (Figure S2)).

Time course analysis of select miRNA and pri-miRNA levels using RT-qPCR revealed that cold treatment increased the level of pri-miRNA171a expression but not other pri-miRNAs tested (Figure 3B). Consistent with the Northern blotting analysis, levels of several miRNA were decreased upon cold treatment regardless of the pri-miRNA levels (Figure 3C). Interestingly, some miRNA levels in *cpl1* were lower than those in wild type even before cold treatment, and remained low during the cold treatment.

The inconsistency in miRNA levels in *cpl1* detected by Northern blotting (maintain untreated WT levels) and RT-qPCR (always low) was recently explained by Manavella et al [45]; RT-qPCR reflects the levels of correctly processed miRNA, whereas Northern blotting detects both correct and incorrect forms of miRNA in *cpl1*. Based on this model, in wild type, cold stress decreases overall miRNA levels, although most of them are correctly processed. Interestingly, miR171a level decreases even its precursor pri-miR171A level increased. This is indicative that processing of pri-miRNA to mature miRNA rather than transcriptional induction of pri-miRNA is a regulatory step for defining miRNA levels during cold stress. This regulation appears impaired in *cpl1*, because correct processing of miRNA is constitutively lower in *cpl1* and total miRNA level remained largely unchanged regardless of the cold stress.

### *cpl1* Interacts with HYL1 and SERRATE

**Figure 4:**
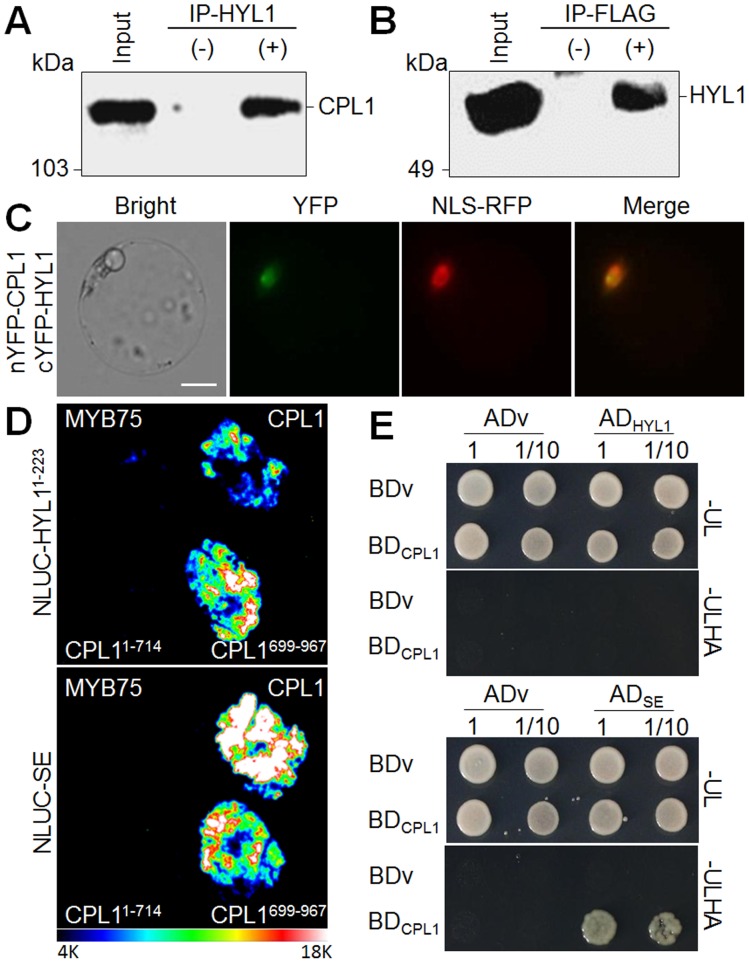
CPL1 interacts with HYL1-SE complex in nucleus. (A) Immunoprecipitation with (+) or without (−) anti-HYL1 was performed using a crude extract of calli containing *gCPL1-FLAG* transgene. CPL1-FLAG was detected by immunoblot using anti-FLAG1 antibody. (B) Immunoprecipitation with (+) or without (−) anti-FLAG was performed using a crude extract of calli containing *gCPL1-FLAG* transgene. HYL1 was detected by immunoblot using anti-HYL1 antibody. (C) BiFC visualization of CPL1-HYL1 interaction. Epifluorescence (YFP) and bright field images of protoplasts that were transfected with plasmids encoding nYFP-CPL1 and cYFP-HYL1 fusion proteins and NLS-RFP. NLS-RFP was used as a positive control for nuclear localization. Yellow signals on merged images indicate co-localization of YFP and nuclear-localized RFP proteins. Scale bars indicate 10 µm. (D) Luminescence images of *N. benthamiana* leaves infiltrated with NLUC-HYL1 (top panel) or NLUC-SE (bottom panel) with CLUC-CPL1 fragments. LUC images were obtained 3 days after infiltration. MYB75, CLUC-MYB75 used as a negative control. (E) Yeast two-hybrid assay. Growth of PJ69-4A co-expressing GAL4 DNA binding domain (BD) fused with CPL1 (BD_CPL1_) and GAL4 activation domain (AD) fused with HYL1 and SE (AD_HYL1_ and AD_SE_). Cells were grown on synthetic dropout (SD) media lacking uracil and leucine (-UL) or SD medium lacking uracil, leucine, histidine and adenine (-ULHA). 2×10^5^ cells were used for (1) and diluted 10-fold for (1/10). Photographs were taken after incubation at 28°C for 48 hours. ADv and BDv indicate vector controls.

The above results indicated that CPL1 is required for proper biogenesis of some miRNAs. Since miRNA are produced from hairpin-shaped dsRNA precursors and CPL1 contains dsRBMs in its C-termini, CPL1 may interact with dsRNA or other dsRBM proteins involved in production of small RNA. However, we did not detect reproducible dsRNA-binding activity in CPL1 dsRBM (data not shown). To test if CPL1 C-terminus functions in protein-protein interaction with dsRBM proteins, we performed co-immunoprecipitation assays using epitope-tagged CPL1 expressed at endogenous level (*gCPL1-FLAG*) and antibodies against dsRBM proteins, namely, HYL1, DRB2, DRB3, DRB4, DRB5, DCL1, DCL3, and DCL4. Bi-directional co-immunoprecipitation successfully detected CPL1 interacting with HYL1, but not with other dsRBM proteins (Figures 4A, B, and data not shown). This interaction was further confirmed by BiFC and LuCI analyses, establishing that the CPL1-HYL1 complex was localized in nuclei (Figures 4C, D), and CPL1^699–957^ containing C-terminal dsRBMs but not CPL1^1–714^ containing the catalytic domain was sufficient. Interestingly, no CPL1-HYL1 interaction was detected using yeast two-hybrid analysis (Figure 4E). By contrast, SE, an interactor of HYL1 [46], could bind to CPL1 with the same specificity to HYL1 *in planta*, and did so in yeast as well (Figures 4D, E). Together, CPL1 forms a complex with HYL1 and SE via the C-terminal dsRBM-containing region, therefore is a part of miRNA producing complex. Interestingly, *cpl1* mutants do not exhibit typical miRNA-deficient phenotype like *hyl1* and *se* mutants, and the expression levels of miRNA target genes were similar to wild type (data not shown). The mild phenotype of *cpl1* plants may be due to overlapping function of CPL1 and its paralog CPL2. The *cpl1 cpl2* double mutant is lethal, perhaps partially due to the lack of essential miRNA/siRNA similar to severe *dcl1* allele [47].

According to the classification of DNA methylation levels in various Arabidopsis RdDM mutants [48], the *cpl1* phenotype likely belongs to “weakly reduced” or “affect only small proportion” category. It is rather surprising that *cpl1* affects DNA methylation and expression levels of RdDM targets since RdDM is generally mediated by siRNA. However, Laubinger et al [24] reported that *dcl1*, which predominantly affects miRNA biogenesis, also affects DNA methylation. Since HYL1 and SE affect DCL1 function [49], it seems likely that weakly reduced DNA methylation in *cpl1 ros1* plants is due to a compromised DCL1-dependent DNA methylation mechanism. Further analyses of target specificity of CPL1-dependent DNA methylation may reveal different branch of DNA methylation pathways.

## Supporting Information

Figure S1 Post-transcriptional silencing of 35S-GUS transgene was intact in *cpl1-2.* GUS activity of 24 -day-old L1 and L1 *cpl1-2* plants were visualized by X-gluc.
(PDF)

Figure S2 Northern blotting analyses of small RNAs in WT and *cpl1* under stress conditions. Two-week-old plants were treated with cold (0°C) for 48 h or with 100 µM ABA for 3 h. *U6* was used as loading controls. The different letters on the right side indicate independently prepared membrane blots.
(PDF)

Table S1 Oligonucleotide primers used in this study.
(PDF)
