# Supplementary figures and images for: Arabidopsis C-Terminal Domain Phosphatase-Like 1 Functions in miRNA Accumulation and DNA Methylation
Source: PLoS One. 2013 Sep 18;8(9):e74739. doi: 10.1371/journal.pone.0074739 (PMC3776750; doi:10.1371/journal.pone.0074739)

**Figure S1.**

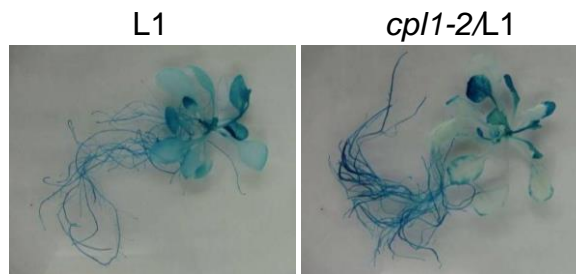

Supplement: Figure S1 — Post-transcriptional silencing of 35S-GUS transgene was intact in cpl1-2. GUS activity of 24 -day-old L1 and L1 cpl1-2 plants were visualized by X-gluc. (PDF) [file pone.0074739.s001.pdf]

**Figure S2.**

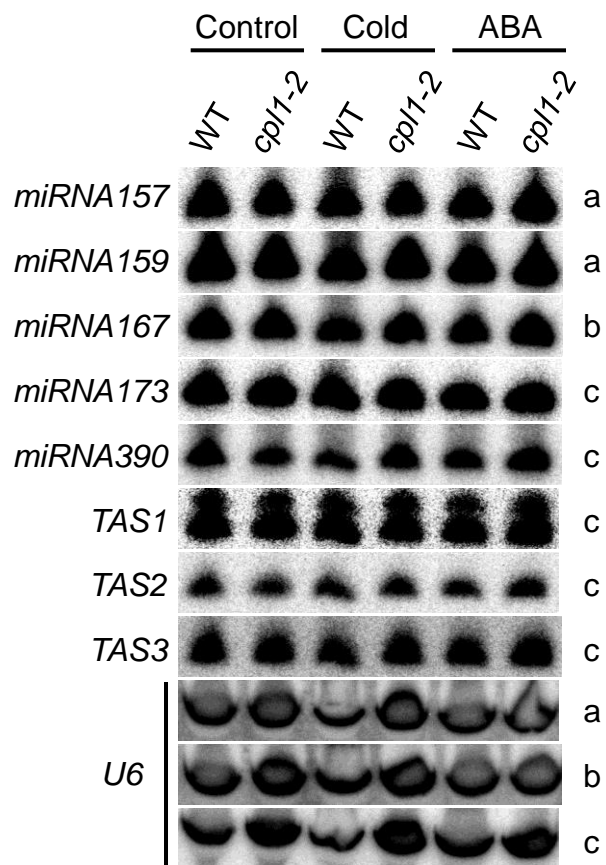

Supplement: Figure S2 — Northern blotting analyses of small RNAs in WT and cpl1 under stress conditions. Two-week-old plants were treated with cold (0°C) for 48 h or with 100 µM ABA for 3 h. U6 was used as loading controls. The different letters on the right side indicate independently prepared membrane blots. (PDF) [file pone.0074739.s002.pdf]
