# Supplementary material for: Arabidopsis C-Terminal Domain Phosphatase-Like 1 Functions in miRNA Accumulation and DNA Methylation
Source: PLoS One. 2013 Sep 18;8(9):e74739. doi: 10.1371/journal.pone.0074739 (PMC3776750; doi:10.1371/journal.pone.0074739)
Supplement: Table S1 — Oligonucleotide primers used in this study. (PDF) [file pone.0074739.s003.pdf]

Table S1. Oligonucleotide primers used in this study.

Oligo name	Sequence	Purpose
LUC F	GCTATTCTGATTACACCCGAG	q-PCR
LUC R	CCTCTGACACATAATTCGCC	q-PCR
AtRD29A F	CAGCACCCAGAAGAAGTTGAACA	q-PCR
AtRD29A R	TCTTGCTCATGCTCATTGCTT	q-PCR
AtCOR15A F	GTTCTCACTGGTATGGCTTC	q-PCR
AtCOR15A R	GATGTTGCCGTCACCTTT	q-PCR
NPT2 F	CATTCGACCACCAAGCGAAACAT	q-PCR
NPT2 R	TGATGCTCTTCGTCCAGATCA	q-PCR
AtCOPIA4 F	CTCACTCAAGCTTCGGTTCC	q-PCR
AtCOPIA4 R	TGTTGGTGAAGGACCGTACA	q-PCR
AtGP1 F	ACAGTGCCACAGTTGAGCAG	q-PCR
AtGP1 R	CAGAAAAATACTCGGTGCCAAT	q-PCR
ATLINE1-4 F	CCGATGGTGACCAAGAGTTT	q-PCR
ATLINE1-4 R	TCAATGTCGGAGACCTCCTC	q-PCR
ENSPM6 F	GACTCGGCGGTGGCTCTAC	q-PCR
ENSPM6 R	TGAGAGACTTACCACTCGGTT	q-PCR
HAT1 F	TCCAAGGCTAGATTTGAGGTCT	q-PCR
HAT1 R	TCACCATAGCGTTGCCCAT	q-PCR
AtMU1 F	GTGGATATACCAAAAACACAA	q-PCR
AtMU1 R	CTTAGCCTTCTTTTCAATCTCA	q-PCR
Helitron1 F	CGCAGATCGTAAGGGTGTTGAT	q-PCR
Helitron1 R	TGGTTAATCCTCTTTAGGTGCCT	q-PCR
Harbinger F	ACCCTGAGTTTAGTTGCTGTCA	q-PCR
Harbinger R	GAGACCACACCCTTATAGCA	q-PCR
ONSEN F	CTT ACC ACA AGA GGA ACC AAC GAA GA	q-PCR
ONSEN R	CTT CGA TCA TGG AAG ACC GGG T	q-PCR
At18SrRNA F	CATAAACGATGCCGACCAG	q-PCR
At18SrRNA R	AGCCTTGCGACCATACTCC	q-PCR
AtACT2 F	TTCCCTCAGCACATTCCA	q-PCR
AtACT2 R	CCCATTCATAAAACCCCAGC	q-PCR
AtTUB8 F	ATAACCGTTTCAAATTCTCTCTCTC	q-PCR
AtTUB8 R	TGCAAATCGTTCTCTCCTTG	q-PCR
RD29A endo, trans Bi F1	AATATTTAGTTTTTTTGTAAATATA	Bisulfite sequencing
RD29A endo, trans Bi F2	ATATGATGGGTTAATAGATATGGAT	Bisulfite sequencing
RD29AendoBi R1	ATAATAATTCCTCTATTTAATCCATTTTCC	Bisulfite sequencing
RD29AendoBi R2	CTAAAATTAAAATCTACCTAAATACTAC	Bisulfite sequencing
RD29AtransBi R1	AAATATTCCACATA CATAATATTCACC	Bisulfite sequencing
RD29AtransBi R2	ATTCTATAATTTATATTCAACCCATATC	Bisulfite sequencing

Oligo name	Sequence	Purpose
AtGP1 F	ACAGTGCCACAGTTGAGCAG	DNA methylation assays
AtGP1 R	CAGAAAAATACTCGGTGCCAAT	DNA methylation assays
AtMu1 F	GTGGATATACCAAAAACACAA	DNA methylation assays
AtMu1 R	CTTAGCCTTCTTTTCAATCTCA	DNA methylation assays
AtSN1 F	ACTTAATTAGCACTCAAATTAAACAAAATAAGT	DNA methylation assays
AtSN1 R	TTTAAACATAAGAAGAAGTTCCTTTTTCATCTAC	DNA methylation assays
RD29A promotor F	GGTGAATTAAGAGGAGAGAGGAGG	Probe amplification for small RNA
RD29A promotor R	GTGGTGGTTCCTCTGTTTGATCCATTTTCC	Probe amplification for small RNA
siRNA1003	ATGCCAAGTTTGGCCTCACGGTCT	Small RNA northern blot
AtREP2	GCGGGACGGGTTTGGCAGGACGTTACTTAAT	Small RNA northern blot
miRNA157a	GTGCTCTCTATCTTCTGTCAA	Small RNA northern blot
miRNA159	TAGAGCTCCCTTCAATCCAAA	Small RNA northern blot
miRNA160a	TGGCATACAGGGAGCCAGGCA	Small RNA northern blot
miRNA161	CCCCGATGTAGTCACTTTCAA	Small RNA northern blot
miRNA164	TGCACGTGCCCTGCTTGTCCA	Small RNA northern blot
miRNA167	TAGATCATGCTGGCAGCTTCA	Small RNA northern blot
miRNA168	TCCCCGACCTGCACCAAGCGA	Small RNA northern blot
miRNA171	GATATTGGCGCGGCTCAATCA	Small RNA northern blot
miRNA173	GTGCTTTCTCTCTGCAAGCGAA	Small RNA northern blot
miRNA390	GGCGCTATCCCTCCTGAGCTT	Small RNA northern blot
TAS1-siR255	TACGCTATGTTGGACTTAGAA	Small RNA northern blot
TAS2-siR1511	AAGTATCATCATTCGCTTGGA	Small RNA northern blot
TAS3-siR2142	GGGGTCTTACAAGGTCAAGAA	Small RNA northern blot
U6	TATGCGTGTCATCCTTGCGCAG	Small RNA northern blot
miR161.1 F	GGC TGA AAG TGA CTA CAT CGG GGT	q-PCR
miR161.2 F	GGG CCT CAA TGC ATT GAA AGT GAC TA	q-PCR
miR164a/b F	AGA AGC AGG GCA CGT GCA	q-PCR
miR168 a/b F	GCT TGG TGC AGG TCG GGA A	q-PCR
miR171a F	GGT GAT TGA GCC GCG CCA ATA TC	q-PCR
pri-MIR164a R	GGG TGA AGA GCT CAT GTT GGA GAA G	q-PCR
pri-MIR164b R	TCA TCA CAC TCA ACA CAC ACA CAC ATC	q-PCR
pri-MIR168a R	TCC GAT TCA GTT GAT GCA AGG CG	q-PCR
pri-MIR168b R	CCT CGG ACT CCG ATT CAG TTG ATA CAA G	q-PCR
pri-MIR171a F	GAG AGT CCC TTT GAT ATT GGC CTG GT	q-PCR
pri-MIR171a R	GCG CGG CTC AAT CAG ATA ATC TAG AGA G	q-PCR
U6 F	TGA CAC GCA TAA ATC GAG AAA TGG TCC A	q-PCR
